# Diaqua­bis­(5-methyl­pyrazine-2-carboxyl­ato-κ^2^ *N* ^1^,*O*)cobalt(II) dihydrate

**DOI:** 10.1107/S1600536811038591

**Published:** 2011-09-30

**Authors:** Qi-Ying Shi, Guo-Chun Zhang, Chun-Sheng Zhou, Qi Yang

**Affiliations:** a Department of Chemistry and Chemical Engineering, Shangluo University, Shangluo 726000, Shaanxi, People’s Republic of China; b College of Chemistry and Materials Science, Northwest University, Xi’an 710069, Shaanxi, People’s Republic of China

## Abstract

In the title complex, [Co(C_6_H_5_N_2_O_2_)_2_(H_2_O)_2_]·2H_2_O, the coordination geometry of the Co^2+^ cation is distorted octa­hedral, with two N atoms and two O atoms from two 5-methyl­pyrazine-2-carboxyl­ate ligands in the equatorial plane. The two remaining coordination sites are occupied by two water mol­ecules. In addition, there are two uncoordinated water mol­ecules in the asymmetric unit. The crystal structure is stabilized by a network of O—H⋯O and O—H⋯N hydrogen-bonding inter­actions, forming a three-dimensional structure.

## Related literature

For related structures, see: Chapman *et al.* (2002 ▶); Fan *et al.* (2007 ▶); Liu *et al.* (2007 ▶); Wang *et al.* (2008 ▶). For their applications, see: Tanase *et al.* (2006 ▶); Ptasiewicz-Bak & Leciejewicz (2000 ▶).
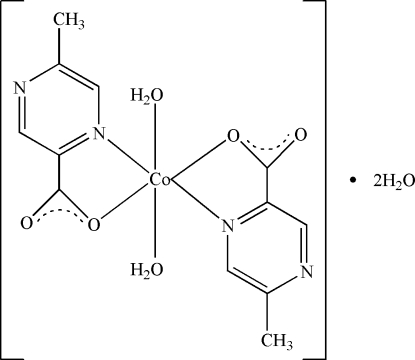


## Experimental

### 

#### Crystal data

[Co(C_6_H_5_N_2_O_2_)_2_(H_2_O)_2_]·2H_2_O
*M* *_r_* = 405.23
Monoclinic, 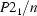

*a* = 10.092 (3) Å
*b* = 13.588 (4) Å
*c* = 12.287 (4) Å
β = 102.961 (6)°
*V* = 1642.1 (9) Å^3^
*Z* = 4
Mo *K*α radiation
μ = 1.10 mm^−1^
*T* = 298 K
0.27 × 0.19 × 0.12 mm

#### Data collection

Bruker SMART APEX diffractometer
Absorption correction: multi-scan (*SADABS*; Sheldrick, 1997 ▶) *T*_min_ = 0.797, *T*_max_ = 0.902
8089 measured reflections
2914 independent reflections
2150 reflections with *I* > 2σ(*I*)
*R*_int_ = 0.030

#### Refinement

*R*[*F* ^2^ > 2σ(*F* ^2^)] = 0.031
*wR*(*F* ^2^) = 0.075
*S* = 1.02
2914 reflections
298 parameters
All H-atom parameters refined
Δρ_max_ = 0.29 e Å^−3^
Δρ_min_ = −0.27 e Å^−3^

### 

Data collection: *SMART* (Bruker, 2002 ▶); cell refinement: *SAINT* (Bruker, 2002 ▶); data reduction: *SAINT*; program(s) used to solve structure: *SHELXS97* (Sheldrick, 2008 ▶); program(s) used to refine structure: *SHELXL97* (Sheldrick, 2008 ▶); molecular graphics: *SHELXTL* (Sheldrick, 2008 ▶); software used to prepare material for publication: *SHELXTL*.

## Supplementary Material

Crystal structure: contains datablock(s) I. DOI: 10.1107/S1600536811038591/ru2012sup1.cif

Structure factors: contains datablock(s) I. DOI: 10.1107/S1600536811038591/ru2012Isup2.hkl

Additional supplementary materials:  crystallographic information; 3D view; checkCIF report

## Figures and Tables

**Table 1:** Hydrogen-bond geometry (Å, °)

*D*—H⋯*A*	*D*—H	H⋯*A*	*D*⋯*A*	*D*—H⋯*A*
O7—H7*WA*⋯N2^i^	0.73 (3)	2.27 (3)	2.940 (3)	154 (4)
O7—H7*WB*⋯O4^ii^	0.91 (4)	2.02 (4)	2.915 (3)	168 (3)
O6—H6*WA*⋯O7^iii^	0.76 (3)	2.09 (3)	2.838 (3)	170 (3)
O6—H6*WB*⋯O2^iv^	0.90 (3)	1.88 (3)	2.780 (3)	173 (3)
O8—H8*WA*⋯N4^v^	0.78 (4)	2.13 (4)	2.861 (4)	156 (4)
O8—H8*WB*⋯O2^vi^	0.72 (3)	2.03 (4)	2.731 (3)	165 (4)
O5—H5*WB*⋯O8	0.76 (3)	1.90 (3)	2.652 (4)	170 (3)
O5—H5*WA*⋯O4^vii^	0.72 (3)	2.02 (3)	2.738 (3)	174 (3)

Symmetry codes: (i) 
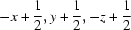
; (ii) 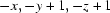; (iii) 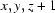; (iv) 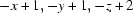; (v) 
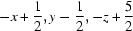
; (vi) 
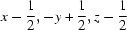
; (vii) 
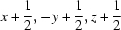
.

**Fig. 1.**
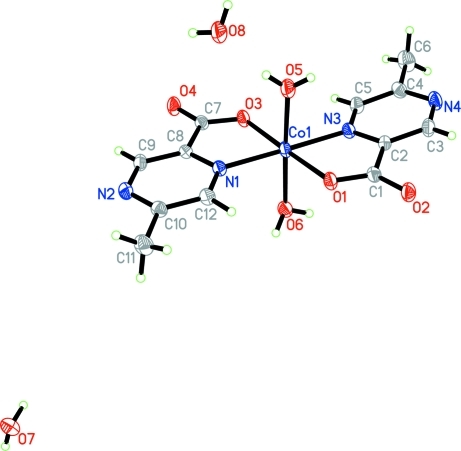
A view of the coordinated environment of the Co2+ atom for complex (I) with the atom-labeling scheme. Displacement ellipsoids are drawn at the 30% probability level.

**Fig. 2.**
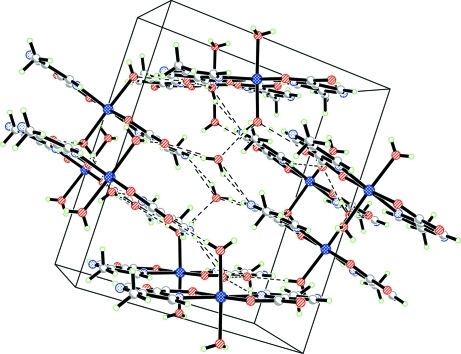
Three dimensional network of the title complex connected through hydrogen bonding.

## supplementary crystallographic information

### Comment

Since the mononuclear complex Cu(mpca)_2_(H_2_O).3H_2_O (Hmpca = 2-methylpyrazine-5-carboxylic acid) was reported by Leciejewicz J. (Ptasiewicz-Bak *et al.*, 2000), many complexes based on the Hmpca have been prepared (Fan *et al.*, 2007; Liu *et al.*, 2007). The complex of Hmpca have been extensively investigated and have often been considered for practical use as a class of functional materials (Tanase *et al.*, 2006). We report here the crystal structure of a Co^2+^ complex, (I)(Figure 1).

Single-crystal analysis shows the complex crystallizes in monoclinic space group *P*2_1_/*n*. As shown in Figure 1, the coordination geometry around Co^2+^ cation can be described a disordered octahedral arrangement with coordination number of 6, where O1, O3, N1 and N3 atoms from two mpca ligands form the equatorial plane, and the axial positions are occupied by O5 and O6 atoms from two coordinated water molecules. Additionally, the complex consists of two uncoordinated water molecules in crystallographic unit. Furthermore, the crystal structure is stabilized by a network of hydrogen-bonding interactions, which O5, O6 atoms from two coordinated water molecules and O7, O8 from two uncoordinated water molecules act as hydrogen-bonding donors to interact with acceptors of O4, O2, N2 and N4 atoms from adjacent ligands, forming a three-dimensional supermolecular structure, as shown in Figure 2.

### Experimental

A mixture of CoCl_2_.H_2_O (0.188 g, 1 mmol), Hmpca (0.304 g, 1 mmol) and distilled H_2_O (8 ml) was sealed in a 23 ml Teflon-lined stainless steel vessel, which was heated at 140° C for 2 days and then cooled to room temperature at a rate of 5°C/h. Red crystals were obtained, washed with ethanol (yield 40% based on Co).

### Crystal data

**Table:**

[Co(C_6_H_5_N_2_O_2_)_2_(H_2_O)_2_]·2H_2_O	*F*(000) = 836
*M**_r_* = 405.23	*D*_x_ = 1.639 Mg m^−^^3^
Monoclinic, *P*2_1_/*n*	Mo *K*α radiation, λ = 0.71073 Å
Hall symbol: -P 2yn	Cell parameters from 3524 reflections
*a* = 10.092 (3) Å	θ = 2.0–25.1°
*b* = 13.588 (4) Å	µ = 1.10 mm^−^^1^
*c* = 12.287 (4) Å	*T* = 298 K
β = 102.961 (6)°	Block, red
*V* = 1642.1 (9) Å^3^	0.27 × 0.19 × 0.12 mm
*Z* = 4	

### Data collection

**Table:**

Bruker SMART APEX diffractometer	2914 independent reflections
Radiation source: fine-focus sealed tube	2150 reflections with *I* > 2σ(*I*)
graphite	*R*_int_ = 0.030
ω scans	θ_max_ = 25.1°, θ_min_ = 2.3°
Absorption correction: multi-scan (*SADABS*; Sheldrick, 1997)	*h* = −10→12
*T*_min_ = 0.797, *T*_max_ = 0.902	*k* = −15→16
8089 measured reflections	*l* = −10→14

### Refinement

**Table:**

Refinement on *F*^2^	Primary atom site location: structure-invariant direct methods
Least-squares matrix: full	Secondary atom site location: difference Fourier map
*R*[*F*^2^ > 2σ(*F*^2^)] = 0.031	Hydrogen site location: inferred from neighbouring sites
*wR*(*F*^2^) = 0.075	All H-atom parameters refined
*S* = 1.02	*w* = 1/[σ^2^(*F*_o_^2^) + (0.035*P*)^2^ + 0.2465*P*] where *P* = (*F*_o_^2^ + 2*F*_c_^2^)/3
2914 reflections	(Δ/σ)_max_ = 0.001
298 parameters	Δρ_max_ = 0.29 e Å^−^^3^
0 restraints	Δρ_min_ = −0.27 e Å^−^^3^

### Special details

**Table:**

Geometry. All e.s.d.'s (except the e.s.d. in the dihedral angle between two l.s. planes) are estimated using the full covariance matrix. The cell e.s.d.'s are taken into account individually in the estimation of e.s.d.'s in distances, angles and torsion angles; correlations between e.s.d.'s in cell parameters are only used when they are defined by crystal symmetry. An approximate (isotropic) treatment of cell e.s.d.'s is used for estimating e.s.d.'s involving l.s. planes.
Refinement. Refinement of *F*^2^ against ALL reflections. The weighted *R*-factor *wR* and goodness of fit *S* are based on *F*^2^, conventional *R*-factors *R* are based on *F*, with *F* set to zero for negative *F*^2^. The threshold expression of *F*^2^ > σ(*F*^2^) is used only for calculating *R*-factors(gt) *etc*. and is not relevant to the choice of reflections for refinement. *R*-factors based on *F*^2^ are statistically about twice as large as those based on *F*, and *R*- factors based on ALL data will be even larger.

### Fractional atomic coordinates and isotropic or equivalent isotropic displacement parameters (Å^2^)

**Table:**

	*x*	*y*	*z*	*U*_iso_*/*U*_eq_	
Co1	0.24651 (3)	0.38090 (2)	0.97992 (3)	0.03100 (12)	
O1	0.44853 (15)	0.40135 (12)	1.05505 (13)	0.0362 (4)	
O3	0.04518 (15)	0.35626 (13)	0.90725 (13)	0.0366 (4)	
O5	0.2709 (2)	0.23572 (17)	1.0370 (2)	0.0473 (6)	
O2	0.59099 (16)	0.44209 (14)	1.21396 (14)	0.0444 (5)	
N1	0.25942 (18)	0.33775 (14)	0.81864 (16)	0.0300 (5)	
O6	0.23198 (19)	0.52962 (15)	0.92703 (19)	0.0396 (5)	
N3	0.23215 (19)	0.42234 (14)	1.14178 (16)	0.0317 (5)	
C1	0.4749 (2)	0.42518 (18)	1.1563 (2)	0.0341 (6)	
N2	0.2339 (2)	0.29075 (15)	0.59604 (17)	0.0387 (5)	
O4	−0.09886 (16)	0.30920 (13)	0.75154 (14)	0.0444 (5)	
C8	0.1372 (2)	0.31958 (16)	0.75246 (19)	0.0295 (5)	
C7	0.0172 (2)	0.32855 (17)	0.8068 (2)	0.0313 (6)	
C10	0.3561 (2)	0.30930 (17)	0.6620 (2)	0.0350 (6)	
C2	0.3548 (2)	0.43145 (17)	1.21038 (19)	0.0318 (6)	
C5	0.1238 (3)	0.42804 (19)	1.1858 (2)	0.0368 (6)	
C9	0.1266 (3)	0.2951 (2)	0.6420 (2)	0.0376 (6)	
C12	0.3680 (3)	0.33191 (18)	0.7746 (2)	0.0348 (6)	
C4	0.1354 (3)	0.43880 (19)	1.2999 (2)	0.0390 (6)	
C11	0.4772 (4)	0.3074 (3)	0.6119 (3)	0.0500 (8)	
N4	0.2575 (2)	0.44717 (16)	1.36849 (17)	0.0445 (6)	
C3	0.3647 (3)	0.4445 (2)	1.3230 (2)	0.0426 (7)	
C6	0.0139 (4)	0.4391 (3)	1.3499 (3)	0.0588 (9)	
O7	0.2904 (2)	0.66789 (18)	0.10443 (19)	0.0497 (5)	
O8	0.2210 (3)	0.06357 (19)	0.9342 (2)	0.0725 (8)	
H7	0.048 (2)	0.2800 (17)	0.599 (2)	0.038 (7)*	
H2	0.042 (2)	0.4240 (17)	1.141 (2)	0.032 (7)*	
H6	0.455 (2)	0.3478 (16)	0.8176 (19)	0.032 (6)*	
H1	0.445 (2)	0.4485 (18)	1.364 (2)	0.040 (8)*	
H10	0.491 (3)	0.368 (2)	0.576 (3)	0.078 (11)*	
H4	0.004 (4)	0.374 (3)	1.382 (4)	0.133 (18)*	
H7WA	0.289 (3)	0.711 (2)	0.069 (3)	0.066 (13)*	
H8	0.556 (4)	0.302 (2)	0.662 (3)	0.080 (12)*	
H5	−0.056 (3)	0.441 (2)	1.306 (3)	0.071 (12)*	
H9	0.475 (3)	0.257 (3)	0.567 (3)	0.075 (11)*	
H3	0.007 (4)	0.494 (3)	1.396 (3)	0.100 (13)*	
H7WB	0.221 (4)	0.673 (2)	0.140 (3)	0.092 (12)*	
H6WA	0.251 (3)	0.561 (2)	0.979 (3)	0.050 (11)*	
H6WB	0.295 (3)	0.540 (2)	0.886 (3)	0.064 (10)*	
H8WA	0.214 (4)	0.021 (3)	0.975 (3)	0.082 (14)*	
H8WB	0.186 (3)	0.052 (2)	0.878 (3)	0.065 (12)*	
H5WB	0.253 (3)	0.190 (2)	1.000 (3)	0.058 (11)*	
H5WA	0.301 (3)	0.224 (2)	1.095 (3)	0.057 (12)*	

### Atomic displacement parameters (Å^2^)

**Table:**

	*U*^11^	*U*^22^	*U*^33^	*U*^12^	*U*^13^	*U*^23^
Co1	0.02774 (19)	0.0445 (2)	0.02001 (18)	−0.00081 (15)	0.00368 (13)	−0.00206 (15)
O1	0.0313 (9)	0.0539 (11)	0.0236 (9)	−0.0024 (8)	0.0064 (7)	−0.0035 (8)
O3	0.0300 (9)	0.0571 (12)	0.0235 (10)	−0.0041 (8)	0.0075 (7)	−0.0046 (8)
O5	0.0590 (14)	0.0465 (14)	0.0291 (13)	−0.0018 (10)	−0.0054 (11)	0.0013 (11)
O2	0.0329 (10)	0.0684 (13)	0.0293 (10)	−0.0099 (9)	0.0012 (8)	−0.0023 (9)
N1	0.0270 (11)	0.0376 (11)	0.0251 (11)	0.0009 (9)	0.0055 (9)	0.0007 (9)
O6	0.0378 (11)	0.0499 (12)	0.0319 (11)	−0.0019 (9)	0.0094 (9)	0.0004 (10)
N3	0.0328 (11)	0.0384 (12)	0.0243 (11)	−0.0001 (9)	0.0071 (9)	−0.0015 (9)
C1	0.0329 (14)	0.0412 (15)	0.0266 (14)	−0.0031 (11)	0.0034 (11)	0.0021 (12)
N2	0.0441 (13)	0.0447 (13)	0.0296 (12)	−0.0051 (10)	0.0134 (10)	−0.0069 (10)
O4	0.0298 (10)	0.0716 (13)	0.0303 (10)	−0.0090 (9)	0.0034 (8)	−0.0097 (9)
C8	0.0337 (14)	0.0301 (13)	0.0247 (13)	−0.0022 (10)	0.0064 (11)	0.0014 (11)
C7	0.0307 (14)	0.0359 (14)	0.0267 (14)	−0.0003 (11)	0.0051 (11)	0.0028 (11)
C10	0.0386 (15)	0.0338 (14)	0.0350 (15)	0.0035 (11)	0.0133 (12)	0.0008 (11)
C2	0.0335 (14)	0.0353 (14)	0.0250 (13)	−0.0048 (11)	0.0034 (11)	−0.0005 (11)
C5	0.0340 (15)	0.0445 (16)	0.0305 (15)	0.0024 (12)	0.0043 (12)	−0.0008 (12)
C9	0.0365 (16)	0.0483 (17)	0.0280 (15)	−0.0085 (12)	0.0074 (12)	−0.0077 (12)
C12	0.0310 (15)	0.0411 (15)	0.0324 (15)	0.0020 (12)	0.0071 (12)	−0.0001 (12)
C4	0.0448 (16)	0.0422 (16)	0.0329 (15)	0.0039 (12)	0.0150 (13)	−0.0007 (12)
C11	0.047 (2)	0.062 (2)	0.046 (2)	0.0041 (16)	0.0222 (16)	−0.0038 (18)
N4	0.0486 (14)	0.0618 (16)	0.0248 (12)	−0.0028 (11)	0.0119 (11)	−0.0055 (11)
C3	0.0395 (17)	0.0593 (19)	0.0270 (15)	−0.0079 (14)	0.0030 (13)	−0.0077 (13)
C6	0.047 (2)	0.088 (3)	0.047 (2)	0.0062 (19)	0.0220 (17)	−0.004 (2)
O7	0.0454 (13)	0.0599 (15)	0.0466 (13)	0.0050 (10)	0.0164 (10)	0.0104 (12)
O8	0.114 (2)	0.0578 (16)	0.0327 (13)	−0.0154 (14)	−0.0111 (14)	0.0038 (13)

### Geometric parameters (Å, °)

**Table:**

Co1—O3	2.0551 (17)	C8—C7	1.514 (3)
Co1—O1	2.0596 (17)	C10—C12	1.396 (3)
Co1—O5	2.090 (2)	C10—C11	1.487 (4)
Co1—N1	2.098 (2)	C2—C3	1.377 (3)
Co1—N3	2.103 (2)	C5—C4	1.388 (3)
Co1—O6	2.118 (2)	C5—H2	0.88 (2)
O1—C1	1.255 (3)	C9—H7	0.88 (2)
O3—C7	1.260 (3)	C12—H6	0.95 (2)
O5—H5WB	0.76 (3)	C4—N4	1.333 (3)
O5—H5WA	0.72 (3)	C4—C6	1.490 (4)
O2—C1	1.247 (3)	C11—H10	0.96 (3)
N1—C12	1.329 (3)	C11—H8	0.89 (4)
N1—C8	1.339 (3)	C11—H9	0.88 (3)
O6—H6WA	0.76 (3)	N4—C3	1.325 (3)
O6—H6WB	0.90 (3)	C3—H1	0.85 (2)
N3—C5	1.326 (3)	C6—H4	0.98 (4)
N3—C2	1.337 (3)	C6—H5	0.79 (3)
C1—C2	1.510 (3)	C6—H3	0.95 (4)
N2—C9	1.330 (3)	O7—H7WA	0.73 (3)
N2—C10	1.339 (3)	O7—H7WB	0.91 (4)
O4—C7	1.244 (3)	O8—H8WA	0.78 (4)
C8—C9	1.378 (3)	O8—H8WB	0.72 (3)
			
O3—Co1—O1	178.22 (7)	O3—C7—C8	115.5 (2)
O3—Co1—O5	91.29 (8)	N2—C10—C12	120.3 (2)
O1—Co1—O5	86.95 (8)	N2—C10—C11	118.5 (2)
O3—Co1—N1	78.99 (7)	C12—C10—C11	121.2 (3)
O1—Co1—N1	101.31 (7)	N3—C2—C3	119.6 (2)
O5—Co1—N1	91.47 (9)	N3—C2—C1	116.0 (2)
O3—Co1—N3	100.57 (7)	C3—C2—C1	124.4 (2)
O1—Co1—N3	79.12 (7)	N3—C5—C4	121.8 (2)
O5—Co1—N3	87.90 (9)	N3—C5—H2	119.1 (15)
N1—Co1—N3	179.22 (7)	C4—C5—H2	119.1 (15)
O3—Co1—O6	91.63 (7)	N2—C9—C8	122.6 (2)
O1—Co1—O6	90.12 (7)	N2—C9—H7	116.4 (16)
O5—Co1—O6	177.05 (9)	C8—C9—H7	121.0 (16)
N1—Co1—O6	89.52 (8)	N1—C12—C10	121.4 (2)
N3—Co1—O6	91.14 (9)	N1—C12—H6	120.7 (14)
C1—O1—Co1	116.60 (15)	C10—C12—H6	117.8 (14)
C7—O3—Co1	117.16 (14)	N4—C4—C5	120.3 (2)
Co1—O5—H5WB	125 (2)	N4—C4—C6	117.9 (3)
Co1—O5—H5WA	122 (3)	C5—C4—C6	121.8 (3)
H5WB—O5—H5WA	113 (3)	C10—C11—H10	113.4 (19)
C12—N1—C8	118.2 (2)	C10—C11—H8	114 (2)
C12—N1—Co1	129.26 (16)	H10—C11—H8	101 (3)
C8—N1—Co1	112.41 (14)	C10—C11—H9	111 (2)
Co1—O6—H6WA	107 (2)	H10—C11—H9	111 (3)
Co1—O6—H6WB	108.3 (18)	H8—C11—H9	106 (3)
H6WA—O6—H6WB	108 (3)	C3—N4—C4	117.2 (2)
C5—N3—C2	117.9 (2)	N4—C3—C2	123.0 (2)
C5—N3—Co1	129.72 (17)	N4—C3—H1	120.2 (17)
C2—N3—Co1	111.75 (14)	C2—C3—H1	116.7 (17)
O2—C1—O1	125.0 (2)	C4—C6—H4	109 (3)
O2—C1—C2	119.0 (2)	C4—C6—H5	114 (2)
O1—C1—C2	116.0 (2)	H4—C6—H5	99 (3)
C9—N2—C10	117.5 (2)	C4—C6—H3	115 (2)
N1—C8—C9	120.0 (2)	H4—C6—H3	117 (3)
N1—C8—C7	115.9 (2)	H5—C6—H3	102 (3)
C9—C8—C7	124.1 (2)	H7WA—O7—H7WB	108 (3)
O4—C7—O3	125.1 (2)	H8WA—O8—H8WB	111 (4)
O4—C7—C8	119.4 (2)		
			
O3—Co1—O1—C1	−78 (2)	C12—N1—C8—C7	−179.3 (2)
O5—Co1—O1—C1	−86.91 (18)	Co1—N1—C8—C7	−2.8 (2)
N1—Co1—O1—C1	−177.79 (17)	Co1—O3—C7—O4	178.88 (19)
N3—Co1—O1—C1	1.54 (17)	Co1—O3—C7—C8	−1.4 (3)
O6—Co1—O1—C1	92.68 (18)	N1—C8—C7—O4	−177.4 (2)
O1—Co1—O3—C7	−100 (2)	C9—C8—C7—O4	3.0 (4)
O5—Co1—O3—C7	−91.29 (18)	N1—C8—C7—O3	2.9 (3)
N1—Co1—O3—C7	−0.04 (17)	C9—C8—C7—O3	−176.7 (2)
N3—Co1—O3—C7	−179.39 (17)	C9—N2—C10—C12	0.2 (4)
O6—Co1—O3—C7	89.15 (18)	C9—N2—C10—C11	−178.5 (3)
O3—Co1—N1—C12	177.6 (2)	C5—N3—C2—C3	1.1 (4)
O1—Co1—N1—C12	−4.2 (2)	Co1—N3—C2—C3	−170.9 (2)
O5—Co1—N1—C12	−91.4 (2)	C5—N3—C2—C1	−179.6 (2)
N3—Co1—N1—C12	−127 (5)	Co1—N3—C2—C1	8.4 (3)
O6—Co1—N1—C12	85.8 (2)	O2—C1—C2—N3	173.5 (2)
O3—Co1—N1—C8	1.64 (15)	O1—C1—C2—N3	−7.6 (3)
O1—Co1—N1—C8	179.85 (15)	O2—C1—C2—C3	−7.3 (4)
O5—Co1—N1—C8	92.67 (16)	O1—C1—C2—C3	171.7 (2)
N3—Co1—N1—C8	57 (6)	C2—N3—C5—C4	−2.8 (4)
O6—Co1—N1—C8	−90.12 (16)	Co1—N3—C5—C4	167.58 (18)
O3—Co1—N3—C5	1.9 (2)	C10—N2—C9—C8	1.4 (4)
O1—Co1—N3—C5	−176.4 (2)	N1—C8—C9—N2	−1.7 (4)
O5—Co1—N3—C5	−89.1 (2)	C7—C8—C9—N2	177.9 (2)
N1—Co1—N3—C5	−53 (6)	C8—N1—C12—C10	1.2 (4)
O6—Co1—N3—C5	93.7 (2)	Co1—N1—C12—C10	−174.60 (17)
O3—Co1—N3—C2	172.67 (16)	N2—C10—C12—N1	−1.5 (4)
O1—Co1—N3—C2	−5.55 (16)	C11—C10—C12—N1	177.1 (3)
O5—Co1—N3—C2	81.75 (17)	N3—C5—C4—N4	2.3 (4)
N1—Co1—N3—C2	117 (5)	N3—C5—C4—C6	−176.2 (3)
O6—Co1—N3—C2	−95.46 (17)	C5—C4—N4—C3	−0.1 (4)
Co1—O1—C1—O2	−178.67 (19)	C6—C4—N4—C3	178.5 (3)
Co1—O1—C1—C2	2.4 (3)	C4—N4—C3—C2	−1.5 (4)
C12—N1—C8—C9	0.3 (3)	N3—C2—C3—N4	1.0 (4)
Co1—N1—C8—C9	176.80 (19)	C1—C2—C3—N4	−178.2 (2)

### Hydrogen-bond geometry (Å, °)

**Table:**

*D*—H···*A*	*D*—H	H···*A*	*D*···*A*	*D*—H···*A*
O7—H7WA···N2^i^	0.73 (3)	2.27 (3)	2.940 (3)	154 (4)
O7—H7WB···O4^ii^	0.91 (4)	2.02 (4)	2.915 (3)	168 (3)
O7—H7WB···O3^ii^	0.91 (4)	2.65 (4)	3.373 (3)	137 (3)
O6—H6WA···O7^iii^	0.76 (3)	2.09 (3)	2.838 (3)	170 (3)
O6—H6WB···O2^iv^	0.90 (3)	1.88 (3)	2.780 (3)	173 (3)
O6—H6WB···O1^iv^	0.90 (3)	2.65 (3)	3.318 (3)	131 (2)
O8—H8WA···N4^v^	0.78 (4)	2.13 (4)	2.861 (4)	156 (4)
O8—H8WB···O2^vi^	0.72 (3)	2.03 (4)	2.731 (3)	165 (4)
O5—H5WB···O8	0.76 (3)	1.90 (3)	2.652 (4)	170 (3)
O5—H5WA···O4^vii^	0.72 (3)	2.02 (3)	2.738 (3)	174 (3)

Symmetry codes: (i) −*x*+1/2, *y*+1/2, −*z*+1/2; (ii) −*x*, −*y*+1, −*z*+1; (iii) *x*, *y*, *z*+1; (iv) −*x*+1, −*y*+1, −*z*+2; (v) −*x*+1/2, *y*−1/2, −*z*+5/2; (vi) *x*−1/2, −*y*+1/2, *z*−1/2; (vii) *x*+1/2, −*y*+1/2, *z*+1/2.
